# Effects of self-monitoring of glucose in non-insulin treated patients with type 2 diabetes: design of the IN CONTROL-trial

**DOI:** 10.1186/1471-2296-10-26

**Published:** 2009-04-27

**Authors:** Uriëll L Malanda, Sandra DM Bot, Piet J Kostense, Frank J Snoek, Jacqueline M Dekker, Giel Nijpels

**Affiliations:** 1 EMGO Institute for Health and Care Research, VU University Medical Centre, Amsterdam, the Netherlands; 2 Department of General Practice, VU University Medical Centre, Amsterdam, the Netherlands; 3 Department of Epidemiology and Biostatistics, VU University Medical Centre, Amsterdam, the Netherlands; 4 Department of Medical Psychology, VU University Medical Centre, Amsterdam, the Netherlands

## Abstract

**Background:**

Diabetes specific emotional problems interfere with the demanding daily management of living with type 2 diabetes mellitus (T2DM). Possibly, offering direct feedback on diabetes management may diminish the presence of diabetes specific emotional problems and might enhance the patients' belief they are able to manage their illness. It is hypothesized that self-monitoring of glucose in combination with an algorithm how and when to act will motivate T2DM patients to become more active participants in their own care leading to a decrease in diabetes related distress and an increased self-efficacy.

**Methods and design:**

Six hundred patients with T2DM (45 ≤ 75 years) who receive care in a structured diabetes care system, HbA1c ≥ 7.0%, and not using insulin will be recruited and randomized into 3 groups; Self-monitoring of Blood Glucose (SMBG), Self-monitoring of Urine Glucose (SMUG) and usual care (n = 200 per group). Participants are eligible if they have a known disease duration of over 1 year and have used SMBG or SMUG less than 3 times in the previous year. All 3 groups will receive standardized diabetes care. The intervention groups will receive additional instructions on how to perform self-monitoring of glucose and how to interpret the results. Main outcome measures are changes in diabetes specific emotional distress and self-efficacy. Secondary outcome measures include difference in HbA1c, patient satisfaction, occurrence of hypoglycaemia, physical activity, costs of direct and indirect healthcare and changes in illness beliefs.

**Discussion:**

The IN CONTROL-trial is designed to explore whether feedback from self-monitoring of glucose in T2DM patients who do not require insulin can affect diabetes specific emotional distress and increase self-efficacy. Based on the self-regulation model it is hypothesized that glucose self-monitoring feedback changes illness perceptions, guiding the patient to reduce emotional responses to experienced threats, and influences the patients ability to perform and maintain self-management skills.

**Trial registration:**

Current Controlled Trials ISRCTN84568563

## Background

Diabetes specific emotional problems can interfere with the strict regime type 2 diabetes mellitus (T2DM) demands. Emotional problems, such as not accepting diabetes, fear for hypoglycaemia and worrying about complications, might impact aspects of quality of life, for example, increase diabetes related distress, which in turn might affect self-care behaviours and glycaemic control [1,2]. Diabetes self-management can protect against the development of diabetes specific distress [3] and it can have a positive effect on perceived self-efficacy [4]. Possibly, common self-management factors such as guidance in accepting diabetes, formulating clear and concrete goals [3] as well as personal confidence and belief in the ability to recognize, understand and act on symptoms related to T2DM may be helpful in reducing levels of distress [5]. An example of a self-management method is glucose self-monitoring. Based on collecting data of glucose levels on different time-points, self-monitoring and its feedback might help patients to a better understanding of day to day variation in glucose levels. With the self-monitoring information, lifestyle adjustments can be made, provided the patient is informed how to interpret the results and what actions to take.

The hypothesis that self-monitoring of glucose empowers the patient by its feedback is based on the principles of Leventhal's self-regulation model [6,7]. This model proposes that individuals construct schematic perceptions of illness and health-threatening conditions according to sources of information that are available to them. These illness perceptions determine how patients respond when confronted with their illness or related threats and are mediators in the willingness and ability to take action. Feedback on the illness condition allows adaption of illness perceptions, which lead to changes in self-efficacy and illness specific distress.

### Previous research

To date, research in self-monitoring of glucose has primarily focused on reaching and maintaining glycaemic control. Therefore, up to now the evaluation of success or failure of self-monitoring has been based upon its ability to decrease HbA1c to normal values.

Self-monitoring of glucose has been proven successful in regulating HbA1c in patients with type 1 diabetes [8] and T2DM requiring insulin [9]. For T2DM patients not using insulin this is still a matter of debate [10-12]. Meta-analyses on self-monitoring of blood glucose (SMBG) in T2DM not using insulin revealed small effects on HbA1c [13-15]. The improvements shown were clinically relevant and statistically significant. Nevertheless, in all reviews the methodological quality of the studies included was questioned. The recently well-designed DiGEM trial addressed most of the methodological comments on the previous studies and found no effects on glycaemic control in T2DM patients not requiring insulin [16].

Before the widespread extent of SMBG, self-monitoring of urine glucose (SMUG) was used to indicate episodes of hyperglycaemia during the corresponding urine excretion time. Even though urine glucose excretion is influenced by renal threshold, SMUG can provide basic information on glycaemic status. Furthermore, feedback derived from SMUG can motivate T2DM patients to become actively engaged in self-management of T2DM [17], irrespective of the monitoring technique. Nevertheless, it is unknown if SMUG provides enough feedback to influence illness perceptions and subsequently diminish diabetes related distress.

Whether self-monitoring of glucose can help patients to better understand their diabetes and to an increased self-efficacy and less diabetes related distress is not known and has not been investigated as a primary objective yet. Information from qualitative research suggest that self-monitoring of glucose may have a negative impact regarding aspects of quality of life and patient-satisfaction [18-20]. Possibly, insufficient knowledge in how and when to perform and interpret SMBG might lead to these results.

In this trial the effect of self-monitoring of glucose regarding diabetes-specific distress and self-efficacy in combination with education in self-monitoring and an algorithm what to do is evaluated.

### IN CONTROL Objectives

The aim of the study is to assess the effects of SMBG and SMUG in patients with type 2 diabetes who are not using insulin compared to usual care. Primary outcome measures are changes in diabetes specific emotional distress and self-efficacy. Secondary outcomes are changes in glycaemic control, patient treatment satisfaction, physical activity, health status, status of depression, occurrence of hypoglycaemia, cost-effectiveness and cost-utility. As part of a process evaluation changes in illness perceptions, perceived severity and social support are determined as well.

## Methods

### Design of the study

"IN CONTROL" entails a 3-armed randomized clinical trial performed in the province of Noord-Holland, The Netherlands. The Medical Ethical Committee of the VUmc approved the study design, protocols, information letters to the patients and informed consent form.

### Setting

The participating diabetes care systems provide and manage regional diabetes care in addition to the care of general practitioners and their nurse practitioners. All care systems are based on the chronic care model [21-23] and coordinate regional diabetes care using a centrally organized database which is available for all involved caregivers. Each patient is annually invited for a physical examination and a visit with a diabetes nurse and a dietician for information, education and advise. This visit includes assessment and discussion of glucose control and the presence of complications. If necessary, follow-up visits are scheduled. The results of the visits are sent to the patients' general practitioner (GP) who is responsible for the management of the patients.

### Study population

In total 600 consenting participants will be recruited. Patients are considered eligible if they are diagnosed with T2DM and meet the following criteria:

❑ known disease duration of over 1 year

❑ recent HbA1c ≥ 7.0%

❑ treated with diet and/or oral hypoglycaemic agents

❑ do not require insulin at inclusion

❑ aged between 45 and 75 years

❑ used SMBG or SMUG less than 3 times in the previous year

**Figure 1:**
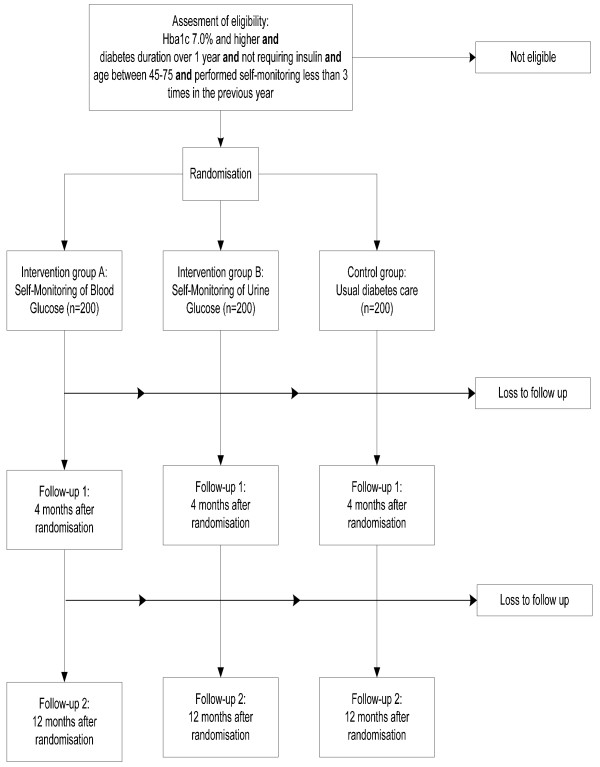
**Design of the RCT**.

Participants will be randomly assigned to one of the following 3 groups (figure 1):

❑ intervention group A, performing SMBG with specific SMBG education, in addition to usual diabetes care provided by the regional diabetes care system.

❑ intervention group B, performing SMUG with specific SMUG education, in addition to usual diabetes care provided by the regional diabetes care system.

❑ control group receiving usual diabetes care provided by the regional diabetes care system.

### Randomization

For intervention allocation a randomization list is drawn up using a computerized randomization computer program (Random Allocation Software version 1.0.0). As different effects may be expected of prescribed drug treatments that are likely to cause hypoglycaemia, for example sulfylureas or glinides (SU), randomization will be pre-stratified by treatment (using SU or not (Non-SU)). A research-manager, who is not involved in the patients care, allocates the patient to one of the 3 groups by using the randomization list. Intervention allocation will be concealed for the principal investigator who is responsible for the data analyses. Participants may withdraw from the study at their own request and without providing reasons. Date of withdrawal will be recorded and effort will be made to contact participants lost to follow up. Only participants withdrawn prior to randomization will be replaced.

### Intervention groups (SMBG & SMUG)

Following randomization participants allocated to one of the intervention groups receive a training in SMBG or SMUG delivered by trained research assistants and a flowchart with instructions when to perform and how to interpret self-monitoring. After this training participants should be able to perform SMBG or SMUG and interpret the results, identify factors that can influence glucose level and identify when additional tests are needed.

The acquired skills in self-monitoring are checked and if necessary corrected in a control visit of maximal 30 minutes, 7 to 14 days after randomization. During this visit a stepwise script outlining the self-monitoring skills needed is used. All participants in the SMBG group are requested to measure their blood glucose twice a week (one weekday, one weekend day) at 6 different time points a day (fasting, 1 1/2 – 2 hrs after breakfast, before lunch, 1 1/2 – 2 hrs after lunch, before diner, before bedtime) with a glucose-meter (LifeScan OneTouch^® ^Ultra^®^2). Participants in the SMUG group are requested to test their urine twice a week (one weekday, one weekend day) in the evening after dinner (Urispec™ plus urine glucose in vitro reagent test strips). Both groups will receive a diary in which they have to record the obtained values for blood or urine glucose. To avoid extra psychological burden we allow participants to adjust self-monitoring to a frequency 'they feel comfortable with' starting from 2 months after baseline until the end of the study.

### Measurements

Outcome measures are obtained at baseline, 4 and 12 months. These are collected by self-administered questionnaires, blood sampling and anthropometry. Diabetes specific distress is assessed by the Problem Areas In Diabetes scale (PAID) [24,25] and self-efficacy by the Confidence In Diabetes Self-care questionnaire (CIDS) [26] adjusted for T2DM. Patient treatment satisfaction is assessed by the Diabetes Treatment Satisfaction Questionnaire (DTSQ) [27], physical activity is assessed by the short International Physical Activity Questionnaire (IPAQ-7) [28], health status and cost utility are assessed with the Euroqol-5D [29-31]. Status of depression is assessed at baseline and at 12 months using the Patient Health Questionnaire-9 (PHQ-9) [32], Demographic variables on age, gender, diabetes duration, marital status and level of education are assessed at baseline by means of a self-administered questionnaire. Glycaemic control is measured by HbA1c-level. Furthermore, BMI and blood-pressure are measured. Participants are asked to register occurrence of hypoglycaemia in care-diaries. Costs of direct health care and indirect non-healthcare will be inventoried in cost-diaries and recorded in 3-monthly intervals. Table 1 summarizes the measures and their timing.

**Table 1:** Study measures

**Measures**	**Baseline**	**2 months**	**3 months**	**4 months**	**6 months**	**9 months**	**12 months**
***Physiological***							
BMI	X			X			X
Blood pressure	X			X			X
HbA1c	X			X			X
							
***Questionnaires***							
PAID	X			X			X
CIDS	X	X		X	X		X
PHQ-9	X						X
DTSQ	X			X			X
Euroqol-5D	X			X			X
IPAQ-7	X			X			X
							
***Process evaluation***							
IPQ-R subscales	X	X		X	X		X
MDQ subscales	X	X		X	X		X
							
***Costs***							
Cost of medication							X
Cost diaries			X		X	X	X

### Process evaluation and quality assurance

A process evaluation will be performed to evaluate our theories of the processes underlying the effect of self-monitoring. For this evaluation changes in mastery are assessed by the subscales personal control and treatment control of the Revised Illness Perception Questionnaire (IPQ-R) [33] and changes in perceptions of diabetes and social support by the subscales perceived severity and perceived social support from a significant other of the Multidimensional Diabetes Questionnaire (MDQ) [34]. These measures are assessed at 5 time-points (baseline, 2 months, 4 months, 6 months and 12 months) (Table 1).

A script outlining the topics to be covered per visit is used to support the nurses and research assistants in their tasks. Before inclusion all research assistants attend a training in self-monitoring (SMBG and SMUG) and before the start of the intervention their self-monitoring skills are tested in a pilot. During the inclusion period the research assistants are audited. Directly after the training the research assistants are asked about their opinions about their acquired skills in self-monitoring and their capability to train and instruct participants following protocol (5-point Likert scale; 1 = fully disagree, 5 = fully agree). This will also be done after the inclusion period. The participants of the intervention groups are asked to give their opinion on 6 items regarding skills, importance and motivation for self-monitoring at baseline and 2 months after baseline (5-point Likert scale).

### Sample size

The proposed trial is designed to detect a clinically relevant change of self-monitoring of glucose on diabetes specific emotional distress and self-efficacy. No consensus exists about minimal important differences (MID) of diabetes specific distress measured with the PAID and self-efficacy measured with the CIDS. Therefore we set the MID at half a standard deviation [35]. In diabetes patients the standard deviation (SD) of PAID (scores transformed to 0–100) was 20 points [25]. Using a bonferroni correction based on two primary hypotheses with α 0.025 and β 0.15, a sample size of 86 per group is needed to find a difference of 10 points (0.5 SD). Information on the SD of the CIDS is not yet available for T2DM patients. For type 1 diabetes patients the SD of CIDS (scores transformed to 0–100) was 12 points [26]. To detect a decrease of 6 points (0.5 SD), while correcting according to bonferroni based on two primary hypotheses with α 0.025 and β 0.15 a sample size of 86 per group is needed. Taking into account different effects in different treatment groups (SU or non-SU) pre-stratification by treatment will double the sample size. Assuming a 15% drop-out rate, a total of 600 T2DM patients with HbA1c ≥ 7.0% are needed in the study. In addition, with a total of 600 patients it is expected to have sufficient power to detect a clinically relevant decrease of 0.5% in HbA1c.

### Analyses

On the basis of an intention-to-treat analysis, differences between the intervention groups and usual care group are calculated with 95% confidence intervals. In addition per protocol analyses will be performed. The subgroups SU and non-SU will be analysed separately for all outcome measures. In case there is no effect modification by treatment the data of both subgroups will be pooled. If there are any relevant differences in baseline measurements between the 3 arms, we will adjust the outcomes for these factors (i.e. age, gender, ethnicity, status of depression, diabetes duration, marital status, level of education). Linear and logistic regression will be used to determine the effect of the intervention on each of the outcome measurements. The effect of the intervention will be estimated for sub-groups defined by duration of diabetes, socio-economic status, perceived social support and perceived severity of diabetes, glycaemic control and self-monitoring frequency in order to gain a better understanding as to who benefits most from the intervention. Analyses of moderators and mediators will be performed for the process-evaluation.

### Economic analyses

For the economic evaluation from both the societal and the health insurer perspective all costs related to diabetes, and diabetic complications are considered relevant. Costs of the intervention (SMBG or SMUG, visits to the GP, medical specialists, therapists, dieticians and hospitalization) are considered direct healthcare costs. The costs of i.e. absence from paid and unpaid work are considered indirect healthcare costs. Differences in mean costs between the groups will be presented with 95% confidence intervals. An incremental cost-effectiveness ratio (ICER) will be given both for PAID and CIDS by dividing the incremental mean costs by the incremental mean reduction in PAID or CIDS score. A cost utility ratio will estimate the incremental costs per Quality Adjusted Life Year gained for the study period. Cost-effectiveness planes and acceptability curves will be presented and 95% confidence intervals will be estimated for the ICERs.

## Discussion

This article presents a detailed description of a RCT, designed to explore the effectiveness of SMBG and SMUG on diabetes specific emotional distress and self-efficacy in non-insulin dependent T2DM patients. The proposed trial is sufficiently powered and, to the best of our knowledge, the first to allow for conclusions on the effect of self-monitoring of glucose on both diabetes specific emotional distress and glycaemic control in a large sample. In addition, due to a pre-stratification in oral treatments likely to cause hypoglycaemia or not, a possible interaction with type of treatment can be discovered. With the use of a theoretical model as a base for our hypothesis the evaluation of processes theoretically underlying the (in)effectiveness of our trial will be allowed as well. Therefore, this trial will make an important contribution to the evidence of self-monitoring in non-insulin requiring patients with T2DM.

Franciosi et al. [36] reported that a self-monitoring frequency of more than 1 measurement a day was related with the development of diabetes related distress in T2DM patients not using insulin. Unfortunately, mastering a new skill is coupled with practicing and thus a relatively high frequency. By offering participants in the intervention groups a training in self-monitoring, a control visit and a flowchart with instructions when to perform and how to interpret self-monitoring we believe that a 2 months period should be sufficient to familiarize with the self-monitoring skills considered necessary. In addition, development of diabetes-related distress caused by a high monitoring frequency is targeted by giving the participants the opportunity to independently adjust the prescribed self-monitoring frequency to one 'they feel comfortable with' starting two months after baseline.

As glycaemic control is an important issue in diabetes care most research regarding self-monitoring has focused on clinically relevant changes in HbA1c [16,37-40]. This trial primarily focuses on the effect of self-monitoring on diabetes specific distress and is additionally sufficiently powered to detect a clinically relevant decrease of 0.5% in HbA1c.

This trial will deliver important insights in the effects of self-monitoring of glucose in T2DM patients not using insulin on diabetes related distress and self-efficacy and the role of possible barriers and facilitators underlying its (in)effectiveness.

## Abbreviations

BMI: Body Mass Index; CIDS: Confidence In Diabetes Self-care questionnaire; DTSQ: Diabetes Treatment Satisfaction Questionnaire; GP: General Practitioner; ICER: Incremental Cost-Effectiveness Ratio; IPAQ-7: short International Physical Activity Questionnaire; IPQ-R: Revised Illness Perception Questionnaire; MDQ: Multidimensional Diabetes Questionnaire; MID: Minimal Important Difference; PAID: Problem Areas In Distress scale; PHQ-9: Patient Health Questionnaire; RCT: Randomized Controlled Trial; SMBG: Self-Monitoring of Blood Glucose; SMUG: Self-Monitoring of Urine Glucose; SU: Sulphonyl-Urea or Glinides; T2DM: Type 2 Diabetes Mellitus.

## Competing interests

The authors declare that they have no competing interests.

## Authors' contributions

ULM is responsible for the data collection and wrote the manuscript. SDMB, JMD and GN developed the original concept for the study. The study design was further developed by ULM, SDMB, PJK, FJS, JMD and GN. All authors have read and approved the final manuscript.

## Pre-publication history

The pre-publication history for this paper can be accessed here:


